# Case Report: UMOD gene mutation and phenotypic overlap with REN in autosomal dominant tubulointerstitial kidney disease

**DOI:** 10.3389/fgene.2025.1661377

**Published:** 2025-12-18

**Authors:** Jingying Xu, Enhui Chen, Wen Shi, Wenhui He, Dongrong Yu, Xianfa Li

**Affiliations:** 1 Department of Nephrology, Zhejiang Chinese Medical University, Hangzhou, China; 2 Department of Nephrology, Hangzhou TCM Hospital of Zhejiang Chinese Medical University (Hangzhou Hospital of Traditional Chinese Medicine), Hangzhou, China

**Keywords:** ADTKD, tubulointerstitial injury, gene mutation, UMOD, REN

## Abstract

Autosomal dominant tubulointerstitial kidney disease (ADTKD) is a rare monogenic kidney disorder characterized by progressive tubular atrophy and interstitial fibrosis. It is primarily associated with pathogenic variants in genes such as UMOD (uromodulin), REN (renin), MUC1 (mucin 1), and HNF1B (hepatocyte nuclear factor 1-beta). We report a unique Chinese case of ADTKD-UMOD in a patient carrying a UMOD gene mutation. The clinical presentation was complex: in addition to the classic features of UMOD mutations (hyperuricemia and gout), the patient exhibited endocrine and metabolic abnormalities typically linked to REN gene defects (ADTKD-REN), including anemia, hypotension, and hyporeninemic hypoaldosteronism. However, renal biopsy and genetic testing ultimately confirmed the diagnosis as ADTKD caused by a heterozygous missense mutation in UMOD gene.

## 1 Introduction

ADTKD (autosomal dominant tubulointerstitial kidney disease) (Eckardt et al., 2015) represents a group of monogenic hereditary kidney disorders characterized by progressive tubulointerstitial damage. It constitutes one of the significant genetic etiologies for end-stage renal disease (ESRD). The clinical phenotype demonstrates high heterogeneity. During early stages, due to insidious clinical manifestations and the absence of specific biomarkers (e.g., urine sediment examination often shows no significant abnormalities), coupled with typically inconspicuous pathological alterations, it is frequently misdiagnosed as chronic kidney disease of unknown etiology. Recent advancements in whole-exome sequencing have significantly improved molecular diagnostic rates for ADTKD. Currently identified pathogenic genes include UMOD (uromodulin gene), REN (renin gene), MUC1 (mucin 1 gene), and HNF1B (hepatocyte nuclear factor 1β gene) (Bolar et al., 2016). Among these, UMOD was the first identified causative gene (ADTKD-UMOD). Its mutations exert dominant-negative effects, leading to abnormal accumulation of misfolded uromodulin in the endoplasmic reticulum of thick ascending limb epithelial cells. This subsequently triggers endoplasmic reticulum stress and cellular apoptosis, ultimately manifesting as early-onset hyperuricemia, urinary concentration defects, and progressive tubulointerstitial fibrosis (Verhave et al., 2016). ADTKD-REN results from mutations in the REN gene encoding preprorenin, with clinical hallmarks including reduced glomerular filtration rate (GFR), hypotension, anemia and gout.

This article presents a case of a Chinese adolescent with ADTKD-UMOD carrying pathogenic UMOD mutations, who paradoxically manifested typical ADTKD-REN phenotypes including hypotension, anemia, and hyporeninemic hypoaldosteronemia, an overlap that is extremely rare and has not been previously reported in the literature.

## 2 Case presentation

A 16-year-old Han Chinese female was hospitalized for persistent hyperuricemia (UA >600 μmol/L) over 4 years and elevated serum creatinine (118 μmol/L) for over 1 year. The patient developed gout onset at age 15 accompanied by hyperuricemia (UA>700 μmol/L), followed by intermittent therapy with sodium bicarbonate and febuxostat. Her medical history indicated mild anemia without menorrhagia, and no known familial predisposition to renal disorders or hyperuricemia was reported.

### 2.1 Clinical and laboratory findings

Physical examination revealed hypotension (85/57 mmHg) with no signs of swelling, joint deformities, or abnormalities in the heart, lungs, or abdomen. Laboratory tests showed mild anemia (hemoglobin 104 g/L; red blood cell count 3.39 × 10^12^/L) with normal erythropoietin levels (8.10 IU/L) and no clinical evidence of gastrointestinal bleeding. Additional findings included elevated serum uric acid (476 μmol/L), impaired kidney function (creatinine 101 μmol/L; eGFR 59.7 mL/min/1.73 m^2^; uric acid clearance 1.34%), and normal potassium levels. Arterial blood gas analysis was unremarkable. Urine tests detected no protein or blood, with 24-h urinary protein was 0.06 g/day. Hormonal testing showed low supine renin (0.05 ng/[mL·h], reference range: 0.15–2.33 ng/[mL·h]) and aldosterone levels (19.92 pg/mL, reference: 28.00–239.00). While upright renin levels fell within the normal range (0.14 ng/[mL·h], reference: 0.10–6.56), upright aldosterone remained abnormally low (19.48 pg/mL, reference: 28.00–376.00). The antinuclear antibody panel was negative. Kidney ultrasound identified shrinkage in both kidneys (left: 9.1 × 4.8 × 3.6 cm; right: 9.0 × 4.9 × 3.6 cm) with uniform tissue texture, consistent with chronic kidney disease. Ultrasounds of the liver, pancreas, and spleen showed no abnormalities.

### 2.2 Renal biopsy and genetic analysis

**FIGURE 1:**
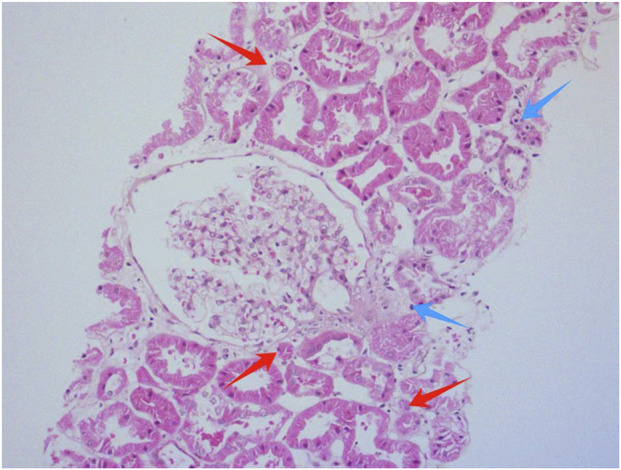
Histological morphology of renal tissue using hematoxylin and eosin (HE) staining. Red arrows indicate focal tubular atrophy; blue arrows point to scattered lymphocytic and mononuclear cell infiltrates in the renal interstitium.

**FIGURE 2:**
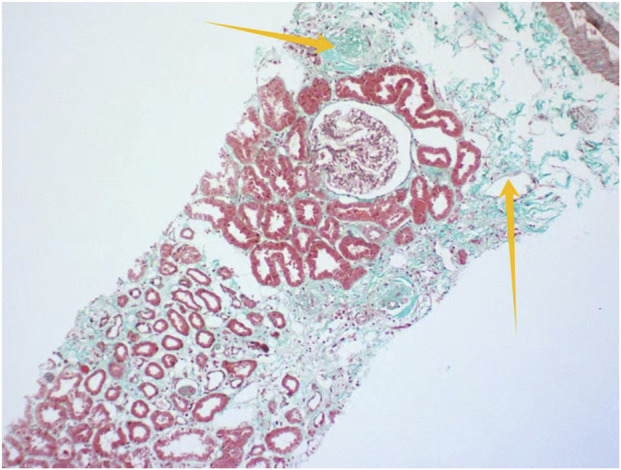
Renal histopathology by MA100 staining (×200) Yellow arrows indicate renal interstitial fibrosis.

**FIGURE 3:**
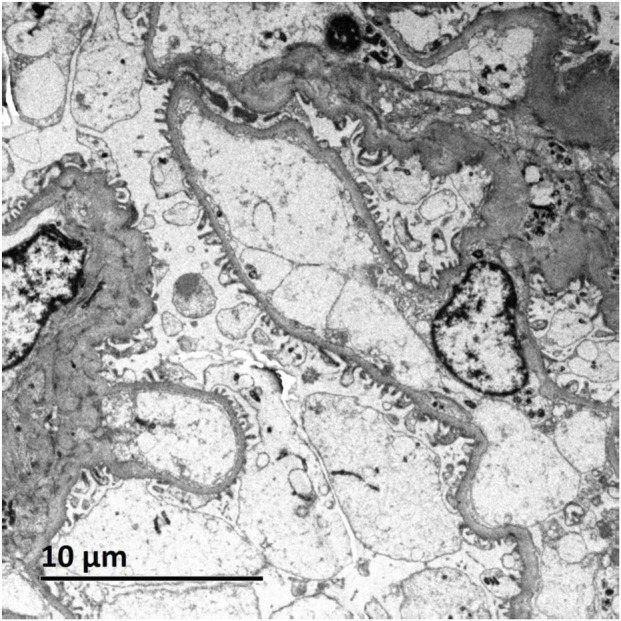
Ultrastructural analysis of renal tissue by electron microscopy (EM).

Light microscopic examination of the renal biopsy specimen revealed 8 glomeruli, including 2 with ischemic sclerosis, 1 globally obsolescent glomerulus, 1 exhibiting cystic transformation, and the remaining glomeruli demonstrating focal segmental mesangial hyperplasia accompanied by segmental endothelial cell pairing and mild mesangial matrix expansion. Additional findings comprised mild interstitial fibrosis (<25%), focal edema, scattered lymphocytic and mononuclear cell infiltrates (<25%), and focal tubular atrophy (<25%), with no significant vascular abnormalities. Immunofluorescence studies were negative for IgA, IgG, IgM, C3, C4, C1q, fibrinogen, κ, and λ light chains, while linear positivity for collagen IV α5 was observed along the glomerular basement membrane. Congo red staining was negative. Electron microscopy confirmed mild mesangial hyperplasia without electron-dense deposits, segmental endothelial cell pairing, intact glomerular basement membranes, and partial foot process effacement, alongside increased lysosomal bodies in tubular epithelial cells (Figures 1–3).

**FIGURE 4:**
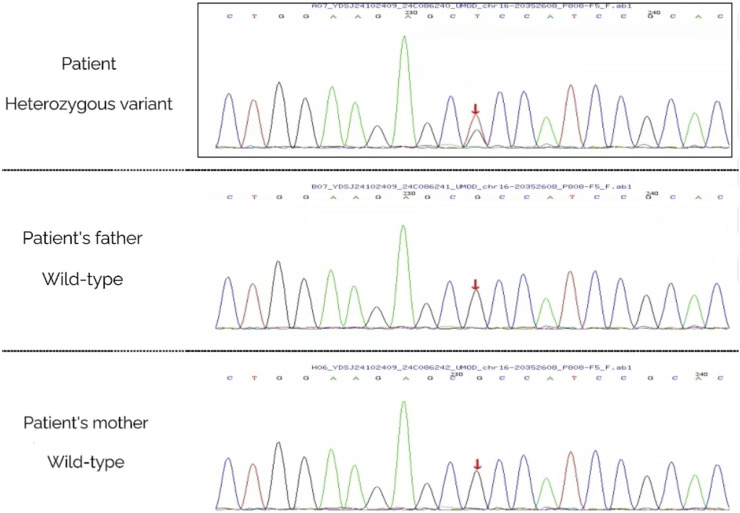
Genetic analysis of the proband and her parents via Sanger sequencing. A (Proband): Red arrow points to UMOD gene c.1382C>A heterozygous mutation (A/G peak) via high-throughput sequencing. B (Father): Red arrow indicates wild-type genotype (C/C peak) at the same locus (no mutation) via Sanger sequencing. C (Mother): Red arrow indicates wild-type genotype (C/C peak) at the same locus (no mutation) via Sanger sequencing.

**FIGURE 5:**
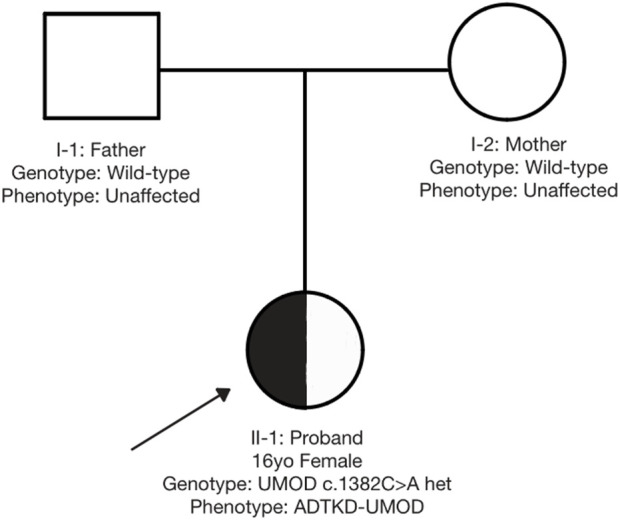
Family pedigree demonstrating *de novo* UMOD mutation. Legend: □ = Male; ○ = Female; ● = Proband. Proband (●): UMOD c.1382C>A heterozygous mutation. Parents (open symbols): UMOD wild-type (no mutation).

Genetic analysis via high-throughput sequencing (MyGenostics, Beijing) identified a heterozygous UMOD missense mutation [c.1382C>A(p.Ala461Glu)]. The familial inheritance pattern was established through parental Sanger sequencing (Figure 4) and is graphically illustrated by the pedigree (Figure 5), which confirms the *de novo* origin of the mutation. This analysis demonstrated that neither parent carried the variant, consistent with the absence of a family history of renal disease or hyperuricemia. Despite phenotypic overlap with ADTKD-REN, targeted reanalysis excluded REN mutations, conclusively attributing the pathology to the UMOD variant. Following the American College of Medical Genetics and Genomics (ACMG) guidelines (PS2+PS4+PM2-Supporting + PP3), this variant was classified as pathogenic, establishing the diagnosis of ADTKD-UMOD.

### 2.3 Management

The patient was prescribed febuxostat (40 mg/d), sodium bicarbonate (0.5 g, three times daily, tid), and increased sodium intake to elevate blood pressure. For adjunctive, personalized renal protection, traditional Chinese medicine (TCM) herbal therapy was additionally incorporated into the treatment plan, guided by the TCM therapeutic strategy of “replenishing qi, activating blood circulation, dispelling dampness, and detoxifying.” Specifically, the herbal prescription included *Astragali Radix* (15 g), *Angelicae Sinensis Radix* (10 g), *Rhei Radix et Rhizoma Praeparata* (3 g), *Pheretima* (wine-fried, 10 g), *Centellae Herba* (30 g), *Persicae Semen* (10 g), and *Smilacis Glabrae Rhizoma* (15 g). This prescription was prepared as a water-based decoction. Patients took one daily dose, which was split into two separate servings. Notably, this Should you have any questions, please contact the corresponding authors.

TCM intervention lacked standardization, as it was customized to the patient’s specific clinical circumstances. Consequently, its reproducibility in other clinical contexts could be restricted.

One month after the patient’s diagnosis, a follow-up was completed. During the follow-up period, there was no recurrence of gout attacks, and no drug-related adverse reactions (such as rash, nausea, or gastrointestinal discomfort) were reported. The patient showed good adherence to the treatment regimen.The long-term follow-up plan is as follows: in the future, serum uric acid, renal function (creatinine, eGFR), and blood pressure will be rechecked every 3 months; renal ultrasound will be rechecked every 6 months; and gene testing will be conducted annually to confirm the mutation carrier status, so as to dynamically monitor the progression of the disease.

## 3 Discussion

Autosomal dominant tubulointerstitial kidney disease (ADTKD) represents a group of monogenic inherited tubulointerstitial nephropathies. Historically, its classification encompassed entities such as familial juvenile hyperuricemic nephropathy (FJHN), medullary cystic kidney disease (MCKD), and renal cysts and diabetes syndrome (RCAD). However, diagnostic confusion and hindered research communication arose due to inconsistent nomenclature. In 2015, the KDIGO guidelines standardized the terminology as “ADTKD” (Eckardt et al., 2015) and established a molecular subtyping system based on causative genes (ADTKD-UMOD, -REN, -HNF1B, -MUC1, and -SEC61A1). This framework not only streamlined diagnostic protocols but also facilitated mechanistic investigations into disease pathogenesis. The KDIGO guidelines emphasize that definitive ADTKD diagnosis requires both genetic evidence (definitive pathogenic mutations) and clinicopathological consistency, with explicit criteria outlined for suspected and confirmed cases. In the present case, despite the absence of a family history, the identification of a UMOD mutation alongside characteristic tubulointerstitial pathological features fulfilled the diagnostic criteria for ADTKD-UMOD.

ADTKD-UMOD is a hereditary renal disorder associated with pathogenic variants in the UMOD gene located at chromosome 16p11.2 (16p11-p13). This gene encodes uromodulin, also known as Tamm-Horsfall protein (THP), a glycoprotein specifically secreted by epithelial cells of the thick ascending limb of Henle’s loop (TAL) and the initial segment of the distal convoluted tubule (DCT) in the kidney. Uromodulin regulates sodium reabsorption through the formation of extracellular matrix networks and participates in innate immune responses (Devuyst et al., 2017). The UMOD gene comprises 11 exons, with exons 2–11 encoding the uromodulin protein. In 2009, Williams et al. identified disease-associated UMOD mutations primarily clustered in exons 3, 4, 5, and 7. Notably, over 95% of reported UMOD mutations localize to exons 3 and 4, predominantly manifesting as missense variants. These mutations disrupt disulfide bonds within the D8C domain, leading to aberrant protein aggregation in the endoplasmic reticulum, activation of the unfolded protein response (UPR), and subsequent tubular epithelial cell apoptosis (Williams et al., 2009). ADTKD-UMOD primarily affects the renal tubulointerstitium, clinically characterized by progressive renal dysfunction, early-onset hyperuricemia/gout, and the absence of proteinuria or hematuria. Paradoxically, the present case exhibited additional features traditionally associated with ADTKD-REN, including hypotension, hyporeninemic hypoaldosteronism, and normocytic anemia, alongside classic manifestations of hyperuricemia and chronic kidney disease (CKD).

ADTKD-REN, previously termed Familial Juvenile Hyperuricemic Nephropathy Type 2 (FJHN2), was renamed to “ADTKD-REN” in accordance with the nomenclature recommendations by KDIGO. The main histopathology shows kidney tubule and interstitium damage. Immunofluorescence reveals decreased or missing prorenin and renin in juxtaglomerular cells and tubular epithelial cells (Bleyer et al., 2010). The disease is caused by mutations in the REN gene located on chromosome 1q32.1, which encodes renin, a protease that cleaves angiotensinogen to generate angiotensin I and thereby initiates the RAAS cascade. Studies suggest that mutations in exon 1 (encoding the signal peptide) may lead to abnormal intracellular accumulation of renin, resulting in apoptosis of renin-producing cells in the glomerular afferent arterioles. However, the exact mechanism by which REN mutations induce tubulointerstitial fibrosis remains unclear. Other clinical manifestations of ADTKD-REN are likely associated with reduced renin secretion and consequent dysfunction of the renin-angiotensin-aldosterone system (RAAS) (Zivná et al., 2009). In ADTKD-REN, heterozygous REN mutations cause decreased renin synthesis, clinically presenting with hypotension, hyporeninemic hypoaldosteronism, anemia, and early-onset hyperuricemia. While ADTKD-UMOD patients typically exhibit normal blood pressure, the present case showed an unusual hypotensive presentation. UMOD encodes uromodulin, and its impaired synthesis or function reduces NKCC2 transporter activity in the thick ascending limb of the loop of Henle, leading to increased urinary sodium excretion (Mutig et al., 2011). However, such alterations can be compensated by a compensatory enhancement of sodium reabsorption in the proximal tubules. Clinically, patients exhibit normal blood volume, with blood pressure maintained within the low-normal to normal range (Zivna et al., 2022). The observed hyporeninemic hypoaldosteronism presents a pathophysiological challenge in this UMOD-related case. Physiologically, the reduction in effective circulating blood volume that would result from sodium wasting is a potent stimulus for RAAS activation. While sodium handling defects in the thick ascending limb characterize UMOD pathology, enhanced proximal tubular reabsorption typically maintains RAAS activation and normal blood pressure in classical ADTKD-UMOD. The unexpected RAAS suppression in our patient therefore represents a fundamental deviation from the expected compensatory response. The early-onset anemia in ADTKD-REN may stem from multiple pathways, including tubulointerstitial fibrosis-induced reduction in erythropoietin (EPO) secretion, renin deficiency due to REN mutations (which lowers angiotensin II levels, a stimulator of erythroid progenitor cells), and chronic inflammation or iron metabolism dysregulation. Notably, while ADTKD-UMOD patients rarely develop early anemia, the current case presented with early anemia despite normal EPO levels, suggesting a potential renin deficiency-related mechanism similar to that in ADTKD-REN.

This case exhibited a complex clinical profile—including hyperuricemia, gout, mild anemia, hypotension, and hyporeninemic hypoaldosteronism—that closely mirrors the phenotype typically associated with ADTKD-REN. However, genetic testing identified a pathogenic heterozygous UMOD mutation, with no REN variant detected. An extensive review of the literature on ADTKD-UMOD case series and reports (Lin et al., 2018; Olinger et al., 2020) confirms that while hyperuricemia and gout are universal, early-onset anemia and significant RAAS suppression are not part of its recognized clinical spectrum. For instance, the large cohort study by Groopman EE et al. described numerous UMOD mutations but did not report such REN-related features (hypotension, hyporeninemic hypoaldosteronism, and anemia) (Groopman et al., 2019). This finding reinforces that genetic testing is essential for definitive diagnosis, whereas clinical phenotypes alone may be misleading. Due to the small sample size, clinical extrapolation of this case is limited and cannot be generalized to all UMOD-mutated patients. Although a rare phenotypic overlap (hypotension, hyporeninemic hypoaldosteronism, anemia) between UMOD mutations and REN-related features was observed, further studies are needed to confirm the overlap’s incidence in ADTKD-UMOD patients and its association with specific mutation sites. Future research could focus on two aspects: first, multicenter collaboration to integrate larger ADTKD-UMOD cohorts, focusing on RAAS indicators and hematological manifestations to clarify the overlap’s epidemiological traits; second, basic experiments to analyze UMOD mutation’s impact on RAAS regulation, providing mechanistic support. This case contributes a new observation to ADTKD-UMOD’s phenotypic heterogeneity rather than establishing a universal conclusion, and more studies are required to refine understanding of the disease’s phenotypic spectrum.

## Data Availability

The raw data supporting the conclusions of this article will be made available by the authors, without undue reservation.
